# Acupuncture treatment of facial spasm: An overview of systematic reviews

**DOI:** 10.1097/MD.0000000000032182

**Published:** 2022-12-16

**Authors:** Yubo Gong, Xuefeng Li, Xue Zhou, Ting Pan, Haili Wang, Chunhai Chen, Jing Wang, Siyi Wang, Xinhua Chen

**Affiliations:** a Changchun University of Chinese Medicine, Changchun, People’s Republic of China; b The Affiliated Hospital of Changchun University of Chinese Medicine, Changchun, People’s Republic of China.

**Keywords:** acupuncture, facial spasm, AMSTAR-2, GRADE, overview, systematic review

## Abstract

To provide evidence, this review evaluated the methodological quality, risk of bias, and reporting quality of SRs/MAs in the treatment of Facial Spasm with acupuncture.

**Methods:**

Systematic reviews and Meta analyses (SRs/MAs) of acupuncture for Facial Spasm were retrieved from 8 databases from inception to October 1, 2022. Two reviewers independently screened the literature and extracted the data, then used Assessment of Multiple Systematic Reviews-2 (AMSTAR-2), Bias Risk in Systematic Review (ROBIS), and Preferred Report Item for Systematic review and Meta–analysis (PRISMA), Grading of Recommendations, Assessment, Development and Evaluation (GRADE) to assess methodological quality, risk of bias, quality of reporting, and quality of evidence.

**Results:**

A total of 8 SRs/meta-analyses were included. All the SRs were published between 2012–2022. Based on AMSTAR-2, 8 SRs were rated critically low quality. By using the ROBIS tool, 6 SRs were rated low-risk bias. With the PRISMA-A checklist, we found 2 out of 8 SRs were found adequately reported over 70%. With the GRADE system, no high-quality evidence was found, and only two outcomes provided moderate-quality evidence. Among the downgraded factors, the risk of bias within the original trials was ranked first, followed by publication bias, inconsistency, and imprecision.

**Conclusion:**

Acupuncture is a promising complementary treatment for HFS. However, due to the low quality of the SRs/MAs supporting these results, high-quality studies with rigorous study designs and larger samples are needed before widespread recommendations can be made.

## 1. Introduction

Hemifacial spasm (HFS) is a common,^[1,2]^ clinically defined movement disorder that is typically characterized by unilateral, involuntary, irregular clonic, or tonic contractions of muscles innervated by the facial nerve.^[3,4]^ Although HFS is not dangerous, it usually causes significant cosmetic and functional disability. HFS may interfere with the patient’s professional and social life and have important health and economic implications,^[5]^ While botulinum toxin is an effective form of management, intermittent relapsing symptoms emerging between treatments can be distressing, and symptoms can be aggravated by a host of external factors, most notably stress. Although microvascular decompression is an effective method for the treatment of HFS, some patients may still refuse this treatment because of its postoperative complications.^[6]^ Worldwide, acupuncture has been accepted as a popular and safe complementary therapy,^[7]^ and it has been widely used to treat HFS by physicians aiming to reduce the side effects of medication and surgery and to increase its therapeutic effectiveness.

Systematic reviews (SRs)/meta-analyses (MAs) are important tools to guide evidence-based clinical practice, and they have been widely used in various medical fields in recent years. However, with the increasing number of SRs/MAs, their quality is uneven, and their conclusions on the same topic of SR/MAs are often contradictory; therefore, the clinical evidence they provide has been criticized. A systematic overview of SRs/MAs is a relatively new approach for synthesizing the outcomes from multiple SRs/MAs, evaluating their quality and attempting to address any inconsistent outcomes. The objective of our study was to critically assess the scientific quality of relevant SRs/MAs regarding the application of acupuncture in the treatment of HFS using a systematic overview.

This is the first overview that comprehensively assessed SRs of acupuncture for HFS with AMSTAR 2, ROBIS, PRISMA-A, and GRADE tools. The objective of this overview is to critically assess the quality of relevant SRs and present an objective and comprehensive evaluation of the effectiveness and safety of acupuncture for HFS, which can help the public and policy-makers understand whether acupuncture should be recommended as a treatment for HFS.

## 2. Materials and Methods

### 2.1. Registration

A predetermined, written protocol of this overview was registered in the PROSPERO (International prospective register of systematic overview) database (https://www.crd.york.ac.uk/PROSPERO/), registration number: CRD42021273334. This overview of SRs/MAs was performed in accordance with guidelines introduced by the Cochrane Collaboration Search Strategy and Preferred Reporting Items for Systematic Reviews and Meta-Analyses (PRISMA) statement.

### 2.2. Inclusion criteria

Type of Studies: This study included SRs/MAs of randomized controlled trials (RCTs) of patients who were diagnosed with facial spasm using definitive diagnostic criteria. Repeated publications and SRs/MAs that were not rigorous were excluded.

Interventions: Studies of acupuncture (e.g.,electroacupuncture, fire needle, or acupuncture ear) or acupuncture plus conventional therapy (CT) as an intervention for facial spasm were included. The control group treatments were CT, medication and placebo.

Outcome Indicators: SRs/MAs should have at least one clear outcome such as the effective rate, cure rate, spastic amplitude, spastic frequency, and intensity of spasticity.

### 2.3. Exclusion criteria:

We excluded studies that met the following criteria: Studies that were published in abstract form with no full text. Non-RCT SRs, commentaries, guidelines, protocols, editorials, and narrative reviews; Duplicated publications of the same SRs.

### 2.4. Data sources and search strategy

Eight electronic databases [Web of Science, The Cochrane Library, PubMed, EMBASE, China National Knowledge Infrastructure (CNKI), Wanfang Database, Sino-Med, and Chongqing VIP] from their inception until August 2, 2021, were searched for potential SRs/MAs, and we conducted an updated search on June 20, 2022, to provide more up-to-date and comprehensive evidence. The search strategies for each database are presented in Supplemental Table 1, Supplemental Digital Content, http://links.lww.com/MD/I74.

### 2.5. Data management and extraction

All articles were read by 2 independent investigators, and data from the articles were validated and extracted according to the predefined criteria. Disagreements between the 2 investigators were resolved through discussion.

### 2.6. Quality assessment

Two independent investigators assessed the methodological quality, reporting quality, risk of bias, and evidence quality by the Assessing the Methodological Quality of Systematic Reviews 2 (AMSTAR-2).^[8]^ Preferred Reporting Items for Systematic Reviews and Meta-Analyses (PRISMA) for the acupuncture checklist,^[9]^ Risk of Bias in Systematic Reviews (ROBIS),^[10]^ and Grading of Recommendations, Assessment, Development, and Evaluation (GRADE),^[11]^ respectively. Disagreements between the 2 investigators were resolved through discussion.

1. The AMSTAR-2^[9]^ is a valid instrument composed of 16 items, including 7 critical items (items 2, 4, 7, 9, 11, 13, and 15), which are used to critically assess the validity of an SR. Each item was evaluated as “yes” (a positive result), “partial yes” (partial adherence to the standard), and “no” (no information is provided to rate an item) according to adherence to the standard.
2. The PRISMA statement is a valid instrument composed of 27 items. Each item was evaluated as “yes,” “partial yes,” and “no,” representing full reports, partial reports, and no reports.^[12]^ The completion of each item is presented as a ratio.
3. The aim of the ROBIS tool is to evaluate the level of bias presented in a systematic review. This tool assesses the level of bias across 4 domains of 2 phases: “study eligibility criteria,” “identification and selection of studies,” “data collection and study appraisal,” and “synthesis and findings.” Each domain has signaling questions and a judgment of concerns about risk of bias of the domain, and the results are rated as “high risk, “low risk, or “unclear risk.”^[10]^
4. The quality of primary outcomes of included SRs was evaluated by the GRADE system. The GRADE system assesses evidence quality with 4 levels: high, moderate, low, or very low. The initial grading would be decreased if there were study limitations, inconsistencies, imprecision, indirectness, or publication bias.^[11]^

## 3. Results

### 3.1. Results on literature search and selection

**Figure 1.**
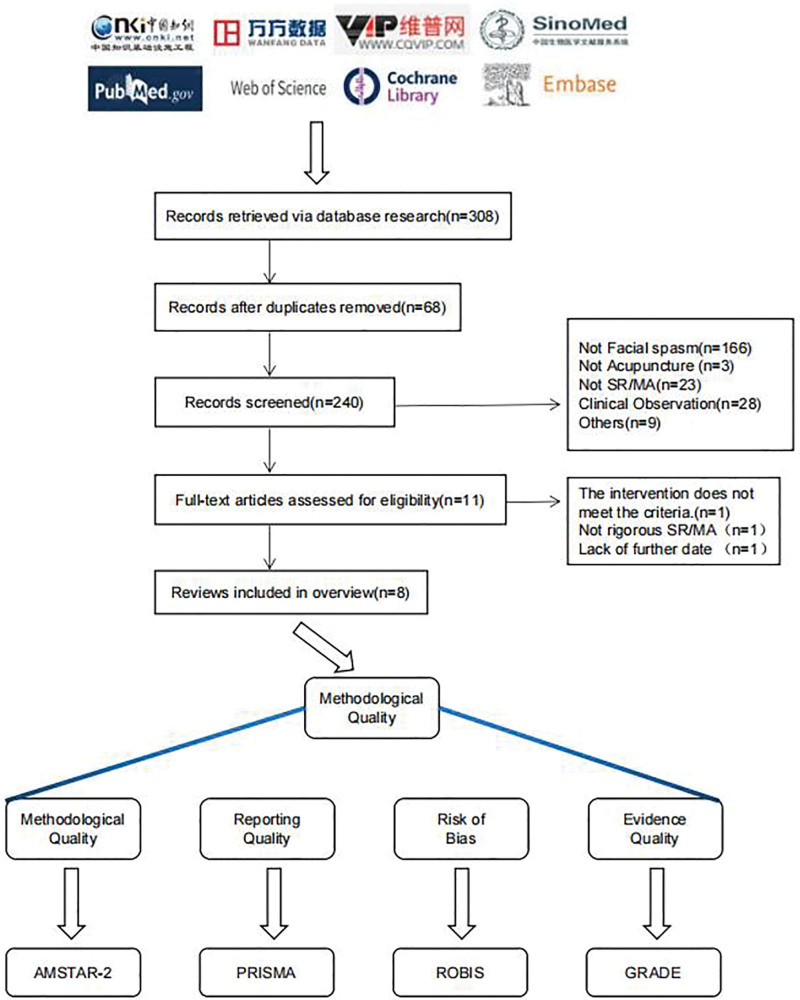
Flow chart for inclusion in literature screening.

In total, 308 publications were retrieved from the 8 databases. After removing duplicates and title/abstract screening, 11 publications were retrieved for full-text assessment. Examining these full-text publications resulted in the exclusion of 3 publications. Finally, 8 publications^[13–20]^ were selected for inclusion in this overview. The screening and selection procedure is presented in Figure 1.

### 3.2. Description of characteristics

The summarized data extracted from the 8 SRs/MAs are presented in Table 1.These included SRs/MAs that were published in the period from 2012 to 2022. Seven of them were written in Chinese,^[13–20]^ and the remaining 1 (20) were written in English. These SRs/MAs were all published by authors from China. The number of RCTs included in these SRs varied widely, ranging from 6 to 17, and the total number of participants ranged from 583 to 1262. Interventions in the therapy group were mainly acupuncture or acupuncture combined with conventional treatment (CT), while CT or sham acupuncture was used in the control group. In terms of the quality assessment scales, one^[20]^ used Jadad, 6^[14–19]^ used the Cochrane risk of bias criteria and one^[13]^ used Cochrane risk of bias criteria combined Jadad scale. All 8 SR/MAs have reached a positive conclusion.

**Table 1:** Main characteristics of the included reviews.

Author, year (country)	Language	Trials (subjects)	Treatment intervention	Control intervention	Quality assessment	Meta-analyses	Outcome measures	Results	Results summary
Qin et al(2021) (China)	Chinese	11 (861)	①;①+③;①+⑤	⑧	Cochrane criteria + Jadad	Yes	Efficiency	The healing rate and apparent efficiency of acupuncture treatment of hemifacial spasm have obvious advantages compared with the western medicine carbamazepine.	Positive
Wu XH et al(2020) (China)	Chinese	13 (936)	①;③;⑤;⑧;①+⑥;①+⑤;①+⑧;①+⑤+⑧	⑤;⑦;⑧	Cochrane criteria	Yes	Efficiency	Acupuncture is better than medicine or fake acupuncture in treating primary hemifacial spasm	Positive
Chen et al(2019) (China)	Chinese	11 (863)	②;①+②;②+⑤;②+⑥	①;③;⑧;①+④	Cochrane criteria	Yes	Efficiency	Fire needle treatment of HFS can be a priority	Positive
Huang JP et al(2018) (China)	Chinese	16 (1154)	③;③+⑤	①;⑧	Cochrane criteria	Yes	Efficiency	Electroacupuncture is an effective method to treat hemifacial spasm, superior to conventional acupuncture and medication	Positive
Song DP (2015) (China)	Chinese	15 (661)	①;①+⑧;①+⑥;③+⑤	⑦;⑧;①+⑧	Cochrane criteria	Yes	Efficiency	Acupuncture combined with other therapies is better than fake acupuncture and medication	Positive
Wang J et al(2013) (China)	Chinese	6 (843)	①;①+⑥;①+③+⑤	⑧	Cochrane criteria	Yes	Efficiency	Acupuncture and its combination therapy are better than drug therapy in treating hemifacial spasm	Positive
Wang Q (2013) (China)	Chinese	17 (583)	①;③;⑤;①+⑥;③+④	⑧	Cochrane criteria	Yes	Efficiency	Acupuncture alone is better than western medicine; acupuncture combined with other therapies is better than western medicine; acupuncture Combination of TCM is better thanTCM treatment; it has not been proved that moxibustion is better than western medicine treatment.	Positive
Wang QP (2012) (China)	English	13 (1262)	①	⑦;⑧	Jadad scale	Yes	Efficiency	Present trials evaluating the efficacy of acupuncture in treatment of facial spasm are mostly poor in methodological quality. These studies showed that acupuncture was superior to other treatments for facial spasm	Positive

①=acupuncture therapy; ②=fire needle; ③=electroacupuncture; ④= scalp acupuncture; ⑤=Moxibustion; ⑥=medication therapy; ⑦=sham acupuncture therapy; ⑧=Conventional treatment (pharmacological treatment such as Carbamazepine, methylcobalamin, and vitamins).

### 3.3. Results of the methodological quality

Considering the methodological quality, all SRs/MAs were regarded as critically low quality because there was more than one critical item that was unmet in the included SRs/MAs. The methodological limitations arose from the following items: item 2 (none of the SRs/MAs registered the protocol), item 7 (none of the SRs explained the reasons for selecting the study type or provided a complete list of excluded studies with reasons), item 13 (3 SRs/MAs did not account for the risk of bias in the primary studies when interpreting the results of the review), and item 15 (4 SRs/MAs did not conduct a publication bias study or discuss its impact on the review). The details are given in Table 2.

**Table 2:**
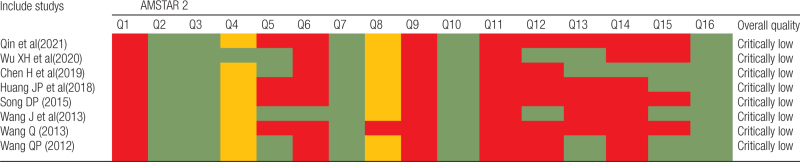
Results of the AMSTAR-2 assessments

### 3.4. Results of the reporting quality

Table 3 presents the overview of the PRISMA for the acupuncture checklist. Some of the papers were of good quality, with an average score of 17.25 and a completion degree of 12.5–21.5 points. The results showed that only 8 items were reported in 100% of all SRs/MAs, and there were still some reporting flaws in other items. In the section of the methods, the topic of the protocol and registration, diagnostic criteria in traditional medicine, search, risk of bias across studies, and additional analyses were reported inadequately (≤50%); in the section of the results, the risk of bias and additional analyses were reported in only 87.5%; in the section of the discussion, limitations were reported in only 12.5%; More details are presented in Table 3.

**Table 3:** Results of the PRISMA for the acupuncture checklist.

Section/Topic	Items	Qin et al(2021)	Wu XH et al(2020)	Chen H et al(2019)	Huang JP et al(2018)	Song DP (2015)	Wang J etal.(2013)	Wang Q (2013)	Song QP (2012)	Yes, n (%)
Title	1. Title	Y	Y	Y	Y	Y	Y	Y	Y	100
Abstract	2. Structured summary	PY	PY	PY	PY	PY	PY	PY	PY	0
Introduction	3. Rationale	Y	Y	Y	Y	Y	Y	Y	Y	100
	4. Objectives	Y	Y	Y	Y	Y	Y	Y	Y	100
Methods	5. Protocol and registration	PY	PY	PY	PY	PY	PY	PY	PY	0
	6. Eligibility criteria	PY	PY	PY	PY	PY	PY	PY	PY	0
	7. Information sources	N	N	N	N	Y	N	Y	N	25
	8. Search	Y	PY	N	PY	Y	N	Y	PY	37.5
	9. Study selection	Y	PY	PY	PY	Y	N	Y	PY	37.5
	10. Data collection process	PY	PY	PY	PY	Y	PY	Y	PY	25
	11. Data items	PY	Y	Y	Y	Y	PY	N	Y	62.5
	12. Risk of bias in individual studies	Y	Y	Y	Y	Y	Y	Y	Y	100
	13. Summary measures	Y	Y	Y	PY	PY	PY	Y	PY	50
	14. Synthesis of results	Y	Y	Y	Y	Y	N	Y	Y	87.5
	15. Risk of bias across studies	N	N	N	N	Y	N	Y	N	25
	16. Additional analyses	Y	Y	Y	PY	Y	N	PY	PY	50
Results	17. Study selection	Y	Y	Y	Y	Y	Y	Y	Y	100
	18. Study characteristics	Y	Y	Y	Y	Y	Y	Y	Y	100
	19. Risk of bias within studies	Y	Y	Y	Y	Y	Y	Y	Y	100
	20. Results of individual studies	Y	Y	Y	Y	Y	PY	Y	Y	87.5
	21. Synthesis of results	Y	Y	Y	Y	Y	Y	Y	Y	100
	22. Risk of bias across studies	N	N	N	N	Y	N	N	N	12.5
	23. Additional analyses	Y	PY	PY	PY	Y	Y	Y	PY	50
Discussion	24. Summary of evidence	N	N	N	N	N	N	N	N	0
	25. Limitations	PY	PY	PY	PY	N	N	N	Y	12.5
	26. Conclusions	N	N	N	N	N	N	N	Y	12.5
Funding	27. Funding	N	N	N	N	N	N	N	N	0
PRISMA score	-	18	17	16.5	16	21.5	12.5	19	17.5	

### 3.5. Results of ROBIS evaluation

For ROBIS, phase 1 assesses the relevance of the research topic, and all SRs/MAs were rated as having a low risk of bias. Domain 1 assessed the study eligibility criteria, and 5 out of 8 SRs/MAs were rated as having a low risk of bias. Domain 2 assessed the identification and selection studies, and no SRs/MAs had a low risk of bias. Domain 3 assessed the collection and study appraisal, and 7 SRs/MAs were at low risk of bias. Domain 4 assessed the synthesis and findings, and no SRs/MAs were rated as having a low risk of bias. Phase 3 considered the overall risk of bias in the reviews, and 6 SRs/MAs were at low risk of bias. More details are presented in Table 4.

**Table 4:** Results of the ROBIS assessments.

Riview	Phase 1	Phase 2				Phase 3
	Assessing relevance	Domain 1: study eligibility criteria	Domain 2: identification and selection of studies	Domain 3: data collection and study appraisal	Domain 4: synthesis and findings	Risk of bias in the review
Qin et al(2021)	☺	☺	☹	☺	☹	☺
Wu XH et al(2020)	☺	☺	☹	☺	☹	☹
Chen et al(2019)	☺	☺	☹	☺	☹	☹
Huang JP et al(2018)	☺	☺	☹	☺	☹	☺
Song DP (2015)	☺	☺	☹	☺	☹	☺
Wang J et al(2013)	☺	☹	☹	☹	☹	☺
Wang Q (2013)	☺	☹	☹	☺	☹	☺
Wang QP (2012)	☺	☹	☹	☺	☹	☺

☺=low risk ☹=high risk.

### 3.6. Evidence quality

Twenty-seven outcomes were evaluated by the GRADE system. According to the evaluation results, no high-quality evidence was found, and only 2 outcomes (7%) provided moderate quality, 14 (52%) were low quality, and 11 (41%) were critically low quality. Limitations (n = 27, 100%) were the most common of the downgrading factors in the included studies. All of the outcome indicators were demoted because of the limitations caused by bias in random, distributive hiding or blind. Followed by publication bias (n = 24, 88%), imprecision(n = 5, 19%), and inconsistency (n = 9, 33%). There were no cases of downgrading due to indirectness. This means that the experimental design of the most included studies had potential bias due to randomization, allocation concealment, or blinding methodologies. The details are given in Table 5.

**Table 5:** Results of evidence quality.

Reviews	Intervention	Outcomes	Limitations	Inconsistency	Indirectness	Imprecision	Publication bias	Relative effect (95% CI)	*P* value	Quality
Qin et al(2021)	① vs.⑧	Effective rate	−1	0	0	0	−1	OR 3.78(2.75,5.19)	<.00001	L
Wu XH et al(2020)	① vs.⑦	Effective rate	−1	0	0	0	0	OR 3.59(1.25,10.31)	<.00001	M
	① vs.⑧	Effective rate	−1	0	0	0	0	OR 4.16(2.45,7.06)	<.05	M
Chen et al(2019)	② vs.①/⑧	Effective rate	−1	0	0	0	−1	OR 2.88(1.97,4.23)	<.00001	L
	② vs.①/⑧	Cure rate	−1	−1	0	0	−1	OR 2.13(1.58,2.86)	<.00001	CL
Huang JP et al(2018)	①vs.③	Effective rate	−1	0	0	0	−1	OR 5.53(2.83,10.81)	<.01	L
	③ vs.⑧	Effective rate	−1	−1	0	0	−1	OR 5.48(2.28,13.13)	<.1	CL
Song DP (2015)	①+⑧ vs.⑧	Effective rate	−1	0	0	0	−1	OR 6.06(1.86,19.67)	<.01	L
	①+⑥ vs.⑥	Effective rate	−1	0	0	0	−1	OR 3.95(1.67,9.35)	<.01	L
	①+④ vs.⑧	Effective rate	−1	0	0	0	−1	OR 14.87(3.74,59.10)	<.001	L
	①+⑤ vs.⑧	Effective rate	−1	0	0	0	−1	OR 3.17(0.66,15.12)	<.05	L
	① vs.⑦	Effective rate	−1	0	0	−1	−1	OR 3.59(1.25,10.31)	<.05	CL
	① vs.⑧	Spastic amplitude	−1	0	0	0	−1	MD 2.77(2.21,3.33)	<.0001	L
	① vs.⑧	Spastic frequency	−1	0	0	0	−1	MD 1.81 (1.15,2.48)	<.00001	L
	① vs.⑦	Intensity of spasticity	−1	0	0	−1	−1	MD 0.64(0.25,1.02)	<.05	CL
Wang J et al(2013)	① vs.⑧	Effective rate	−1	0	0	0	−1	OR 7.74 (4.84,12.24)	<.00001	L
Wang Q (2013)	① vs.⑧	Effective rate	−1	0	0	0	−1	OR 4.89 (3.31,7.23)	<.0001	L
	①+⑥ vs.⑥	Effective rate	−1	0	0	0	−1	OR 14.17(4.45,45.10)	<.0001	L
	⑤ vs.⑧	Effective rate	−1	−1	0	−1	−1	OR 1.99(0.81,4.90)	<.1	CL
	① vs.⑧	Spastic amplitude	−1	−1	0	−1	−1	SMD 0.76(0.34,1.19)	<.001	CL
	① vs.⑧	Spastic frequency	−1	−1	0	−1	0	MD 0.68(−0.12,1.47)	>.05	CL
Wang QP et al(2012)	① vs.④	Effective rate	−1	−1	0	0	−1	RR 1.47(1.14,1.91)	<.001	CL
	① vs.⑧	Effective rate	−1	0	0	0	−1	RR 1.37(1.28,1.47)	<.001	L
	①+⑥ vs.⑥	Effective rate	−1	−1	0	0	−1	RR 2.47(1.64,3.72)	<.001	CL
	① vs.④	Cure rate	−1	−1	0	0	−1	RR 2.27(1.73,2.97)	<.001	CL
	① vs.⑧	Cure rate	−1	0	0	0	−1	RR 2.07(1.60,2.68)	<.001	L
	①+⑥ vs.⑥	Cure rate	−1	−1	0	0	−1	RR 4.00(2.11,7.58)	<.001	CL

① = acupuncture therapy; ② = fire needle; ③ = electroacupuncture; ④=massage; ⑤ = Moxibustion; ⑥ = Chinese medicine therapy; ⑦ = sham acupuncture therapy; ⑧ = Conventional treatment (pharmacological treatment such as Carbamazepine, methylcobalamin and vitamins) -1, downgrade; 0,not downgrade; CL,critically low; L,low; M,moderate; H,high; MD, mean difference; RR, relative risk/risk ratio; OR, odds ratio;

### 3.7. The effectiveness of acupuncture for HFS

According to the moderate-quality evidence, the effective rate of acupuncture was superior to western medicine (odds ratio (OR) = 4.16, 95% confidence interval (CI) = (2.45, 7.06), *P < *.05). The effective rate of acupuncture was superior to sham acupuncture therapy (OR = 3.59, 95%, CI = (1.25, 10.31), *P < *.00001).^[14]^ Besides, acupuncture was more effective in reducing HFS amplitude (MD = 2.77, 95%, CI = (2.21, 3.33), *P* < .00001), spastic frequency (MD = 1.81, 95%, CI = (1.15, 2.48), *P* < .00001) and spastic intensity (MD = 0.64, 95%, CI = (0.25, 1.02), *P* < .05) than western medicine and sham acupuncture.^[17]^

### 3.8. The safety of acupuncture for HFS

Of all the 8 SRs, 5 SRs^[13,15,17–19]^ mentioned the adverse events of acupuncture in the treatment of HFS. 3 SRs^[13,15,18]^ did not further analyze the safety evaluation due to the small number of studies. 2 SRs^[17,19]^ recorded adverse events in the control group and no adverse events occurred in the acupuncture group, indicating that acupuncture is a safe therapy for HFS. Recorded adverse reactions occurred in the control group, and no adverse reactions occurred in the acupuncture group, indicating that acupuncture is a safe treatment for HFS.

Ethics and dissemination: The protocol for this systematic review did not require ethical approval because it did not involve humans. This article will be published in peer-reviewed journals and presented at conferences.

## 4. Discussion

### 4.1. Summary of main findings

This is the first overview of SRs that investigate the effectiveness and safety of acupuncture for HFS. We rigorously appraised the published SRs with AMSTAR 2, ROBIS, PRISMA-A, and GRADE. Based on AMSTAR 2, 8 SRs were rated critically low quality. By using the ROBIS tool, 6 SRs were rated low-risk bias. With the PRISMA-A checklist, we found 2 out of 8 SRs were found adequately reported over 70%. The results of GRADE suggested that acupuncture is an effective and safe method for HFS.

First, almost all SRs/MAs did not register a protocol, which may result in a larger adjustment of the study process than expected, increasing the risk of bias and affecting the rigor of the systematic review. Second, 3 of 8 SRs/MAs provided search keywords and specific search strategies, likely contributing to making the comprehensiveness of the literature search difficult to ensure. Third, all included SRs/MAs did not provide a list of excluded trials with reasons for exclusion, which may undermine the transparency of the SRs/MAs and affect the reliability of their results. Furthermore, the included SRs/MAs have different degrees of shortcomings in the reasonable explanation of bias risk, the data synthesis process, publication bias, and funding support information, which affects the quality of SRs/MAs and reduces the utility of the evidence.

Second, for GRADE, the risk of bias was the most common (27/27, 100%) downgrading factor in the included SRs/MAs, which means that the original trials included in the SRs/MAs were of poor quality. Assessing the methodological quality of the original RCTs, most of them refer only to randomization and do not provide a random sequence generation method; most of the RCTs do not explicitly state that treatment allocation was concealed; only a few RCTs mentioned blinding, and most of the subjects and doctors were not blinded. Well-designed and implemented RCTs are considered the gold standard for evaluating interventions to minimize or avoid bias.^[12]^ Therefore, when the quality of the included RCTs is unsatisfactory, the risk of bias increases and may ultimately affect the authenticity of the results of SRs/MAs. Furthermore, it is worth noting that although most of the included SRs/MAs indicated that acupuncture appears to be an effective treatment for HFS. However, there is still the problem of small sample size and imprecise experiments. Therefore, more high-quality RCTs with large sample sizes are essential to determine whether acupuncture is beneficial for HFS.

Third, all of the SRs/MAs included in this overview were conducted in China, and no unpublished studies using patients of different races were found, which may lead to a risk of publication bias. The included SRs/MAs were published in both Chinese and English languages, and the articles published in both languages contained positive results. No significant risk of publication bias was found in the Chinese and English language publication forms. Acupuncture is currently used to relieve HFS symptoms in many clinics in the West as well as the East, but there has been little research on its effectiveness; thus, this may affect the application of the results to an international population. Further studies on this topic should be carried out in both the East and the West in the future.

### 4.2. Implications for future research

Alone or as an adjunct to other interventions, acupuncture has been effectually used in the clinical practice of treating patients with HFS. To improve the reliability of study conclusions and direct clinical practice, future studies should focus on the following points: the reviewer should register or publish the study protocol in advance to avoid any risk of bias and to ensure the rigor of the SR/MA process; in the process of article screening, each step should be recorded in detail, and a list of excluded studies and reasons for exclusion should be listed; Funding sources should be mentioned in the reviews because the results of business-funded studies might be biased toward the funder; (4) studies on this topic should be carried out in both the East and the West in the future.

### 4.3. Strength and limitations

As an overview of acupuncture for HFS, this study can provide a comprehensive evidence reference for clinical practice. The evaluation process through AMSTAR-2, PRISMA, ROBIS, and GRADE revealed obvious limitations in SRs/MAs and RCTs, which may help guide future high-quality studies. However, it is also limited since the evaluation of quality is a subjective process, and different authors may have their own judgment on each factor, so the results may be different from other reviews, although our overview has been evaluated and checked by 2 independent authors. There might be some missing information since we only gathered studies in English and Chinese. We were unable to synthesize all the evidence, which may decrease the accuracy of the conclusions.

## 5. Conclusions

This overview suggests that acupuncture is a promising complementary treatment for HFS. However, the current SRs/MAs are greatly limited by low methodological quality. Therefore, researchers should improve the scientific research design of future acupuncture RCTs for HFS to improve their quality and better guide clinical treatment.

## Author contributions

All authors made a significant contribution to the work reported, whether that is in the conception, study design, execution, acquisition of data, analysis and interpretation, or in all these areas; took part in drafting, revising or critically reviewing the article; gave final approval of the version to be published; have agreed on the journal to which the article has been submitted; and agree to be accountable for all aspects of the work.

**Conceptualization:** Yubo Gong, Chunhai Chen.

**Data curation:** Siyi Wang, Ting Pan, Haili Wang.

**Formal analysis:** Xuefeng Li, Yubo Gong.

**Funding acquisition:** Xinhua Chen.

**Investigation:** Siyi Wang, Ting Pan.

**Methodology:** Yubo Gong, Chunhai Chen.

**Supervision:** Xinhua Chen.

**Validation:** Xuefeng Li, Xue Zhou, Jing Wang.

**Writing—original draft:** Yubo Gong.

**Writing—review and editing:** Yubo Gong, Chunhai Chen, Xinhua Chen.

## Supplementary Material
