# Supplementary material for: Acupuncture treatment of facial spasm: An overview of systematic reviews
Source: Medicine (Baltimore). 2022 Dec 16;101(50):e32182. doi: 10.1097/MD.0000000000032182 (PMC9771242; doi:10.1097/MD.0000000000032182)
Supplement: Supplementary file 1 [file medi-101-e32182-s001.pdf]

Table A Search strategies of PubMed database

Strategies	
#1	Acupuncture [Mesh]
#2	Acupuncture [ti, ab] OR fire needle [ti, ab] OR Electroacupuncture [ti, ab] OR electric stimulation therapy [ti, ab] OR acupuncture therapy [ti, ab] OR acupuncture points [ti, ab] OR acupuncture ear [ti, ab] OR auriculotherapy [ti, ab] OR Acupressure [ti, ab] OR Moxibustion [ti, ab]
#3	(#1 OR #2)
#4	Hemifacial Spasm [Mesh]
#5	Facial spasm [ti, aFacial spasmb] OR Unilateral Facial Spasm [ti, ab]
#6	(#4 OR #5)
#7	Facial Paralysis [Mesh]
#8	Hemifacial Paralysis [ti, ab] OR Bell Palsy [ti, ab]
#9	(#7 OR #8)
#10	Meta-analysis[Mesh]
#11	Systematic [ti, ab] OR Review [ti, ab] OR Meta-analysis [ti, ab]
#12	(#10 OR #11)
#13	(#6 OR #9)
#14	(#3 AND #12 AND #13)

Table B Full text articles excluded with reasons

Full text articles excluded	Reasons
Zhou XX (2018)[1]	The intervention does not meet the criteria.
Peeraully T (2013)[2]	Not rigorous SR/MA
Jo NY (2013)[3]	Lack of futher date

1 Zhou XX. Observation and Meta-analysis of neuroendoscopy-assisted MVD in the treatment of hemifacial spasm [D]. Qingdao University, 2018.

2 Peeraully T, Hameed S, Cheong PT, et,al.. Complementary therapies in hemifacial spasm and comparison with other movement disorders. Int J Clin Pract. 2013 Aug;67(8):801-6.

3 Jo NY, Kim JH, Roh JD. Clinical review of the effects of hominis placental pharmacopuncture in the treatment of facial spasm patients. J Pharmacopuncture. 2013 Sep;16(3):52-7.
